# Spatiotemporal Variations of Plague Risk in the Tibetan Plateau from 1954–2016

**DOI:** 10.3390/biology11020304

**Published:** 2022-02-13

**Authors:** Xing Yuan, Linsheng Yang, Hairong Li, Li Wang

**Affiliations:** 1 Key Laboratory of Land Surface Pattern and Simulation, Institute of Geographic Sciences and Natural Resources Research, Chinese Academy of Sciences, Beijing 100101, China; yuanx.19b@igsnrr.ac.cn (X.Y.); yangls@igsnrr.ac.cn (L.Y.); wangli@igsnrr.ac.cn (L.W.); 2 College of Resources and Environment, University of Chinese Academy of Sciences, Beijing 100049, China

**Keywords:** Himalayan marmot, plague natural foci, climate change, spatiotemporal distribution, Tibetan Plateau

## Abstract

**Simple Summary:**

Climate variability has influence on plague outbreaks worldwide. Usually, plague cases increase with increasing precipitation. Currently there are many studies on the epidemics of plague in human beings, whereas there are few studies on the dynamic of plague in animal. Nevertheless, animal plague is key in the natural epidemiological cycle of plague. We identified spatiotemporal changes of the plague territories in the Tibetan Plateau only using animal plague records. Our risky plague maps are far superior to the county-based maps used currently and have valuable applications for directly informing conservation and management decisions locally and regionally.

**Abstract:**

Plague persists in the plague natural foci today. Although previous studies have found climate drives plague dynamics, quantitative analysis on animal plague risk under climate change remains understudied. Here, we analyzed plague dynamics in the Tibetan Plateau (TP) which is a climate-sensitive area and one of the most severe animal plague areas in China to disentangle variations in marmot plague enzootic foci, diffusion patterns, and their possible links with climate and anthropogenic factors. Specifically, we developed a time-sharing ecological niche modelling framework to identify finer potential plague territories and their temporal epidemic trends. Models were conducted by assembling animal records and multi-source ecophysiological variables with actual ecological effects (both climatic predictors and landscape factors) and driven by matching plague strains to periods corresponding to meteorological datasets. The models identified abundant animal plague territories over the TP and suggested the spatial patterns varied spatiotemporal dimension across the years, undergoing repeated spreading and contractions. Plague risk increased in the 1980s and 2000s, with the risk area increasing by 17.7 and 55.5 thousand km^2^, respectively. The 1990s and 2010s were decades of decreased risk, with reductions of 71.9 and 39.5 thousand km^2^, respectively. Further factor analysis showed that intrinsic conditions (i.e., elevation, soil, and geochemical landscape) provided fundamental niches. In contrast, climatic conditions, especially precipitation, led to niche differentiation and resulted in varied spatial patterns. Additionally, while increased human interference may temporarily reduce plague risks, there is a strong possibility of recurrence. This study reshaped the plague distribution at multiple time scales in the TP and revealed multifactorial synergistic effects on the spreading and contraction of plague foci, confirming that TP plague is increasingly sensitive to climate change. These findings may facilitate groups to take measures to combat the plague threats and prevent potential future human plague from occurring.

## 1. Introduction

In history, plague pandemics have had devastating effects on politics, economics, and demographics [1]. Plague is caused by the bacteria *Yersinia pestis*, which is directly transmitted via human to human or rodent to human contact or via fleas [2]. *Yersinia pestis*, hosts, and its vectors are essential components for plague transmission and only exist in specific geographical conditions (plague natural foci) [3,4]. Changes in climate and land use affect land surface vegetation and landscape patterns of the foci, resulting in the dispersal, migration, and adaptation of the species (hosts and vectors) and impacting the risk dynamics of plague transmission [5].

The Tibetan Plateau (TP) is a major plague area in China and is highly sensitive to climate change. Since the mid-1950s, there has been a significant warming trend in the TP [6], which has made the ecological environment of local foci unstable and has resulted in changeable and complex plague dynamics [7]. An analysis of the molecular genetics of *Y. pestis* in the Qinghai Plateau (a significant part of the TP) found that *Y. pestis* populations have shifted spatially because of local niche changes [8]. Indeed, since 1954, over 687 thousand km^2^ in 88 counties have gradually become threatened by plague in the TP. And 96.8% of plague cases were caused by Himalayan marmot (main plague hosts in the TP). In this marmot plague foci, animal plague pandemics continuously and human plague outbreaks occur frequently [9]. According to statistics, more than 3502 *Y. pestis* were isolated between 1954 to 2020, among which 95.26% were from infected animal and vectors, 4.68% were from human and 0.06% were from soil. In particular, *Y. pestis* has been isolated from animals for 65 years. Besides, more than 65% of *Y. pestis* were isolated in Qinghai Province and Tibet Autonomous Region. To date, at least 24 species of mammals and 13 species of fleas have been identified as infected by *Y. pestis* in the foci, meaning that the risk of contact with any species of infected animal can be substantial [8]. In recent years, the tourism industry of the TP has grown significantly, which further increases the spread risk of plague. However, the current reported plague risk areas are based on a county scale, which makes it difficult for public managers to allocate surveillance resources more reasonably and to achieve effective prevention and control efforts. Consequently, it is essential to identify the truly threatened plague territories to implement a more effective focus control.

Ecological niche models (ENMs) allow the assessment of how plague risks respond to changing environments and provide spatially explicit risk maps of plague [10,11,12,13,14]. Many studies have attempted to understand plague outbreak dynamics using ENMs and some general patterns of factor-attributable risks have been made. These models have demonstrated good predictive ability and have helped identify underlying spatial patterns and reveal certain qualitative connections [15,16]. However, most previous studies focused on the relationships between human plague cases, species abundance, and climate variation [17,18]. Hence, the following problems still exist: (1) Humans are accidental hosts and are not part of the maintenance cycle of plague. The transmission of plague is the result of a complex and multifactorial system and only using human case data in models without considering host ecology will inevitably lead to areas with high human density or connectivity being identified as high-risk regions [19]. (2) When reservoir species (one part of the epizootic cycle) are considered, they are not sufficient to clarify the landscape epidemiology and infection ecology of plague [10,20]. Likewise, if only climate variables are considered, but biotic factors are ignored, the range and niche may produce erroneous inferences [21]. (3) There is a mismatch between the scale of existing climate data and the scale at which organisms experience their environment. Many prior models do not incorporate spatiotemporal heterogeneous ecological and environmental information, such as environmental differences, leading to variation in host suitability. Previous studies tended to select the mean effects of climate over multiple years, generally an average of 50 years [10,22]. However, there is a lack of detailed studies on the historical spatiotemporal dynamics of risk and on the driving factors of risk under different climates. Therefore, these studies may not be sufficient to disentangle variations in plague enzootic foci, diffusion patterns, and their possible links [23].

In this study, we aimed to: (1) identify the finer scale plague regions to break through the currently zoned foci on the county scale; (2) estimate time-series changes of the identified plague regions to reveal the mechanism between the incidence of plague and the changing environment. More specifically, We propose a time-sharing strategy which has the following advantages: (1) only plague data isolated from animals were used, which can minimize contingency; (2) environmental variables of ecological significance beyond climate surfaces were selected by considering *Y. pestis*, hosts, and vectors, which can improve the definition of ecological niche and habitat properties; (3) the plague data were matched by dividing them into five groups according to epidemic characteristics with the meteorological and vegetation data of the same period, which is helpful for matching temporal consistency. From all these data, five separate models from different times were built to define the geographical limits of plague foci as well as their changes. And their predominant influencing factors were identified in each model to clarify the feedbacks of spatial variation under changing climate.

## 2. Materials and Methods

### 2.1. Plague Data Compilation

Epidemic data for *Y. pestis* were compiled from published literature in the History of Plague Epidemic in China, Plague Prevention and Control in Tibet for 50 Years and Qinghai Plague. There were 1607 *Y. pestis* isolated in animals and 531 *Y. pestis* isolated from vectors from 1954–2016 in Qinghai Province and Tibet Autonomous Region. However, we can hardly direct exact geo-location for each *Y. pestis* from the constrained description, so we used infected regions data which were well documented as the spatial scale of village or hamlet. Then we transformed each occurrence site into spatial points using Google Earth and ArcGIS 10.8 software (ESRI, Redlands, CA, USA). By all the infected regions, we removed duplicates when they occurred in the same hamlet and year. Finally, 623 valid geographic points were compiled as epidemic data that applied in this paper.

### 2.2. Environmental Variables of Plague Foci

Twenty-two ecophysiologically relevant variables were initially selected as factors that play a significant role in plague occurrence. The explanations behind variable choice and generation of data layers are available in Table 1 and Table S1. Generally, as far as the hosts are concerned, their density, distribution and migration are closely related to vegetation conditions. Vegetation growth depends on climatic factors which have indirect effects on hosts. In addition, seasonal variation of vegetation phenology may make hosts emerge earlier in the spring from hibernation. As for fleas, meteorological factors, especially temperature, play a key role on their life cycle, infection rate, bacterial virulence and so on. As for *Y. pestis*, although it remains controversial, it is certain the strains exist steadily in specific natural foci after a long period of natural selection and evolution. We hence selected soil and topography to delineate finer plague ranges.

Five types of data were collected: topography, vegetation, soil, climate, and river network data. Digital Elevation Model (DEM) data were obtained from the GMTED2010 dataset (Global Multi-resolution Terrain Elevation Data 2010 courtesy of the U.S. Geological Survey) in Google Earth Engine (GEE) [24]. The river network data calculated by DEM can be downloaded directly in RESDC [25], which was then used to calculate Euclidean distances in ArcGIS 10.8. Gravity data were provided by the Technical University of Denmark [26]. The vegetation data were also from GEE in the dataset of LANDSAT3/5/7/8 (GLS1975, LANDSAT SR from courtesy of the U.S. Geological Survey) [27]. Subsequently, normalized difference vegetation index (NDVI) was calculated and the median values of each pixel were exported into five stages: 1958–1979 (hereafter, S1), 1980–1989 (hereafter, S2), 1990–1999 (hereafter, S3), 2000–2009 (hereafter, S4), and 2010–2016 (hereafter, S5). The geochemical landscape data were derived from the Atlas of Plague and its Environments in the People’s Republic of China [28]. Detailed classification of the geochemical landscape was in Table S2. The pH in H_2_O at a depth of 200 cm was obtained from GEE in the OpenLandMap dataset [29]. Soil data were obtained from Harmonized World Soil Database (HWSDv1.2), and FAO90 attributes were used. Their detailed list can be found on pages 24 and 25 of the PDF [30]. Soil moisture and climate data were obtained from TerraClimate in GEE [31] and were then processed as mean values for the above five stages. Meanwhile, the minimum monthly temperature values were also obtained between April and October (Table 1). The reason we chose the above time scale was the consistency of the historical plague prevalence [8].

In addition, global terrestrial Human Footprint maps for 1993 and 2009 [32] were used to clarify the influence of human disturbance by grouping values based on their changes. Using the results from Section 3.2, we combined human footprint maps to determine the relationship between human activity and the spatial variation characteristics of plague areas. We calculated the difference in human footprint between 1993 and 2009 and divided it into six levels based on its changes. Moreover, the average risk and the percentage of areas with risk values greater than 0.5 for different levels of human disturbance were calculated.

Most noticeably, highly correlated variables may result in over-fitting and hinder obtaining the actual response curves. Hence, we excluded redundant variables that had a high correlation (R ≥ |0.8|) determined by Pearson’s correlation analysis [42,43] in R4.0.4 (Figure S1). The most important variables selected are summarized in Table 1. All environment variables were projected onto Albers-CGCS2000 coordinate system. In particular, to avoid spatial mismatch between the size of organisms and the scale at which climate data were collected, we resampled all the variables to a spatial resolution of 5 km [44], which is the migration distance of marmot. Therefore, we regarded a 5-km spatial resolution as the basic area unit for plague infection among animals.

### 2.3. Modelling the Potential Areas of Animal Plague

Maxent quantifies the statistical relationship between predictor variables at locations where a species has been observed versus background locations in the study region. This software has been proven to perform the best in the potential species distribution assessment [45,46,47].

We divided the valid set of plague occurrence points into five subsets according to years (S1–S5). For each period, 10 models were established to guarantee that the results were stable, and every model was created with the occurrence localities by randomly selecting 75% of the occurrence localities as training data with cross-validation, reserving the remaining 25% for testing. The ultimate outcomes were selected from an average of 10 replicates. In addition, we assigned a combination of four types of features to generate models, including L (linear), Q (quadratic), P (product), and H (Hinge) features. We used default values in all runs, that is, threshold = 10^−5^, maximum iterations = 1000 for the algorithm convergence, and regularization value β = 1. The default regularization value was applicable for this study because we did not involve model transfer across either space or time. Each pixel in the study area was assigned a nonnegative probability value using Maxent. We chose the logistics outputs for easier usage and interpretation, which gave an estimate between 0 and 1 for risk of presence. The threshold segmentation was determined by the “maximum training sensitivity plus specific logic threshold” method: all pixels with a value greater than the threshold were classified as risk areas of plague [12,48]. Based on the classified results, the average risks over the TP and the total areas of risk regions were analyzed.

The model evaluation was first verified to perform significantly better than random [45]. Considering that we need to compare performance from different stages, the area under the ROC curve (AUC), which has been proved useful to compare performance between multiple Maxent models, was used to measure performance between the five averaged models. The closer the values are to 1.0, the better is the performance of the model. Models with values above 0.75 were generally considered potentially useful [46]. The importance of variables was evaluated using the Jackknife [49].

## 3. Results

### 3.1. Model Performance and Changes of Plague Areas

Each average from the five stages of the 10 replicate models had high AUC scores (>0.9) and consistently performed significantly better than random across the entire spectrum (Table 2). The results suggested our five-stage models had high performance, and the estimated distributions were a close approximation of the real probability distribution.

The predicted risk area increased by 17.7 thousand km^2^ from S1 to S2, decreased by 71.9 thousand km^2^ from S2 to S3, increased by 55.5 thousand km^2^ from S3 to S4, and finally decreased by 39.5 km^2^ from S4 to S5 (Table 2). The average risks showed similar fluctuation trends (Table 2). Consequently, S2 and S4 were regarded as stages of increased plague risk, but the risk decreased in S3 and S5.

Moreover, we compared our results with the officially announced areas of confirmed foci counties. The area of confirmed foci counties across the study period continually increased from 99.79 thousand km^2^ in S1 to 687.04 thousand km^2^ in S5, which were almost twice as large as our predicted areas in S3 and S4 and 2.6 times as large as that in S5 (Table 2).

### 3.2. Plague Risk Areas at Different Time

**Figure 1:**
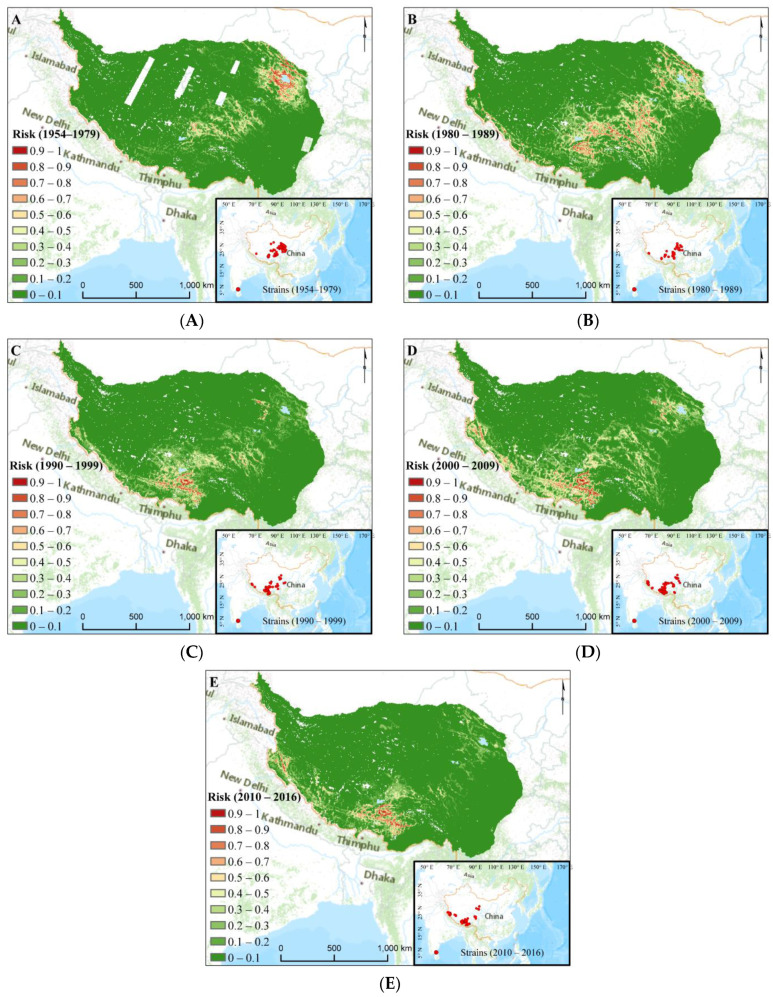
Distributions of plague risk at various stages. (**A**) The distributions of plague risk in 1954–1979 (White blocks are due to the scarcity of Landsat data with cloud cover less than 10); (**B**) The distributions of plague risk in 1980–1989; (**C**) The distributions of plague risk in 1990–1999; (**D**) The distributions of plague risk in 2000–2009; (**E**) The distributions of plague risk in 2010–2016. Furthermore, most of the historical plague data shown in the bottom right corner has been anonymized by aggregation at the county level, fulfilling rules of confidentiality.

Figure 1 shows the identified plague territories of the five stages, and each of the five niches estimated almost exactly coincided with their respective occurrence points. Here, we were mainly interested in pixels that had a higher risk value than 0.5 to analyze spatial variation. The estimated ranges exhibited significant inter-decade variability from 1954–2016. Most noticeably, very high (risk > 0.7) and high (risk > 0.5) risk regions differed significantly between stages in both location and scope.

In the initial stage (S1), very high-risk regions tended to cluster around Qinghai Lake, the northern Qilian area, as well as Gonghe, Xinghai counties in Tibetan Autonomous Prefecture of Hainan and Nagqu area in northeastern Tibet (Figure 1A). In S2, the high-risk regions spread and were heavily located in the southwest TP; in particular, there were many zones in and near the south of the Tibetan Autonomous Prefecture of Yushu in southern Qinghai (Figure 1B). Simultaneously, some regions in Lhasa and the surroundings were at higher risk. In S3, plague risk throughout Qinghai Province was reduced (Figure 1C). However, in S4, a handful of scattered high-risk regions appeared in the south of the Delingha, Wulan county, Dulan county in Haixi Mongolian, and Yushu city in Tibetan Autonomous Prefecture in Qinghai Province (Figure 1D). In particular, Maxent prediction identified the middle part of the TP as an area of constant high risk for the geographical range of the plague. Specifically, the risk regions were larger in central and southern Tibet plague and were prolonged over a long period from S2 to S5 (Figure 1B–E).

Overall, we identified abundant discontinuous niches for plague over the entire TP, and large differences in risk patterns were apparent from 1954–2016. The areas underwent repeated spreading and contraction of spatial ranges. Spreading occurred in S2 and S4 while contraction occurred in S3 and S5.

### 3.3. Impacts of Environmental Variables in Different Time

**Figure 2:**
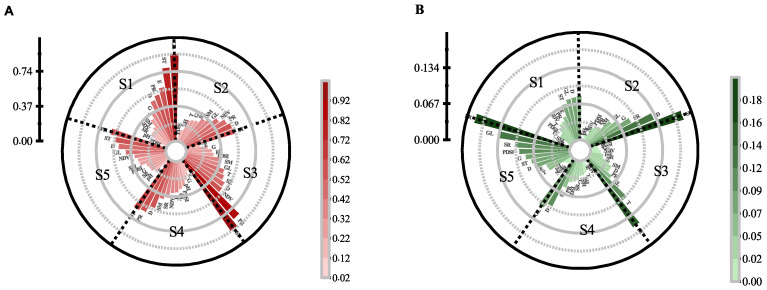
The results of the Jackknife test of variable importance. (**A**) The training gains of each variable if the model was run in isolation, and the variable had useful information when the gain was high. This is useful for identifying the variables that contribute the most individually; (**B**) The reduction in gains when the variable is excluded compared to all variables. If it reduces the gain most when it is excluded, the variable has unique information. Additionally, intrinsic features include GL, ST, D, pH, E, and G; variable features include PR, SR, SM, NDVI, PDSI, and T.

To determine the factors that led to the characterization of distributed differences, we analyzed the alteration of variable importance in each stage. The variable importance dynamics were analyzed using the jackknife test. The quantified results are depicted in Figure 2 where higher values of gain represent a greater contribution when training the models. The changing spatial patterns are profoundly associated with different factor combinations. Intrinsic features had definitive advantages in fitting the distribution of occurrence data in S1. Because these variables contained more useful information, the top three in order were GL (0.93), ST (0.78), and E (0.58). However, in the subsequent stages, ST had displaced GL, and it did not change until PR contributed the highest gain in S5 (Figure 2A). After that, there came to at least one variable feature in the first three rankings instead of merely intrinsic variables since S2. Notably, two variable features appear in S3. More specifically, in S3, the highest gains were ST, followed by PR (0.83), and NDVI (0.52). For S2, S4, and S5, ST, D, and PR had relatively uniform gains (even distribution around 0.5–0.6), but different sorting results.

In general, the importance of the primary variables and other factors changed continuously across the study period, which resulted in diverse distributions of plague between stages. The main drivers shifted from solely predominant invariable attributes (elevation and geochemical landscape) to include both invariable and variable attributes (soil and rainfall). In this conversion, variable importance analyses revealed an increasing explanation of precipitation for plague, with diffusion along a precipitation gradient (Figure 2B). In addition, relatively uniform gains in the combined variables corresponded to wider spatial distributions (Figure 2B). In contrast, the spatial ranges tended to shrink when each variable had different gains (Figure 2A). However, on the whole, variables including ST, D, GL, PR, and their combinations were always key factors for plague risk. They not only showed some of the strongest relationships (Figure 2A), but also contained unique information (Figure 2B). Similarly, NDVI was an important variable that corresponded to the plague risk distribution.

### 3.4. Human Disturbance Evaluation on the Different Distributions

Our modelling environmental variables were natural factors; however, human factors, including urbanization and construction projects, could have altered the environment of some regions during the study period.

The mean values showed a decreasing trend under different degrees of human disturbance (Table 3), indicating that human activities had some restraining effects on plague intensity (Figure S2). And the differences were highly significant from S2 to S3. Further statistics on the high-risk areas (>0.5) showed that these areas decreased steeply following human disturbance, but then increased after the change in human footprint was greater than 2 (Table 3). The results revealed that the plague risk has been controlled by human activity to a certain extent since S3. However, with increasing human activity, the plague risk has gradually returned to pre-disturbance conditions.

## 4. Discussion

Plague regions of TP were identified using models, which represented the finer and true risky areas better. Because in the current publications, plague risk zonation and management were based on counties. And the risk areas were manually estimated based on the sizes of townships. In addition, the estimates are annual accumulations failing to consider plague dynamics, especially dynamics for decreasing risk. Besides, we found that the most evident indicator of Himalaya plague foci was the existence of independent foci with scattered distributions in the vast geographic regions over the TP. Indeed, the range edges, extents, and clustering of foci exhibited spatiotemporal variations caused by different climate variable combinations and human activity.

### 4.1. Basic Factors for Plague Distributions

**Figure 3:**
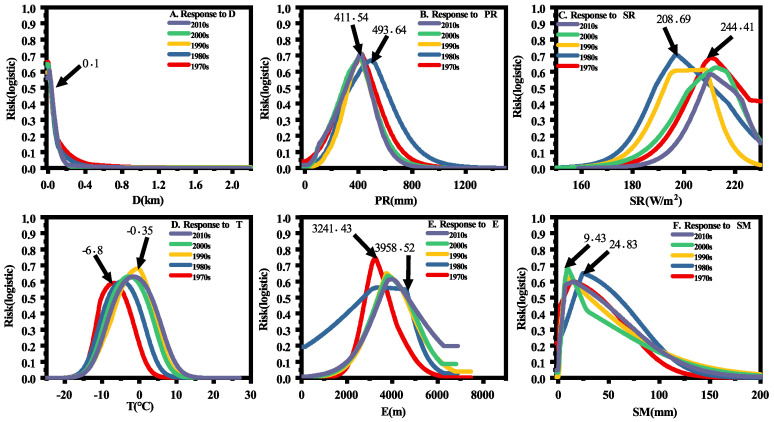
Response curves of environmental variables. The response curves were derived from Maxent runs with variables used in isolation to avoid interference with other variables. They describe how the logistic prediction responds with alteration of each environmental variable. (**A**) The response curves of distance to river in different time; (**B**) The response curves of rainfall in different time; (**C**) The response curves of solar radiation in different time; (**D**) The response curves of temperature in different time; (**E**) The response curves of elevation in different time; (**F**) The response curves of soil moisture in different time.

The variables of intrinsic features are important delineators of the risk distribution of plague. The risk ranges tended to be in a basic niche when intrinsic features were the main factors impacting distribution. This contributed much to the qualitative analysis of foci, because the delineated ranges were closely correlated with the natural focus characteristics of the plague. For example, geochemical landscape and soil type, which had the highest gains in S1 and S3 (Figure S3), showed a wide border with risk regions. Our analysis indicated that high-risk regions were predicted in soils rich in calcium, and this result is compatible with the conclusion of previous studies that plague occurs more easily in calcium soils. In addition, we found that plague tended to occur in leptosols and gleysols, particularly in gelic gleysols [16], where excessive soil moisture accumulates during seasonal melts. The regions with lighter texture and shallower soil layers were ideal for marmots to dig deep burrows and were beneficial for keeping the burrows dry owing to their strong infiltration capacity [50]. Therefore, these soil types are essential and *Y. pestis* can persist for years in these soil conditions [37]. In that sense, the plague natural foci were concentrated in these areas. Similarly, the distance to a river was a relatively strong and useful predictor across all stages and the risk of plague was higher closer to rivers (Figure 3A). Habitat preference may explain the close relationship between risk and D as infected hosts often suffer from dehydration as a symptom and need access to water to relieve discomfort; therefore, they are easier to find closer to rivers.

In addition, the contribution of elevation continuously decreased, reducing from 0.58 to 0.1, indicating that E had less impact on risk distribution (Figure 3E). The response curves of E showed that the highest risk occurred in S1 at an altitude of 3241.43 m, however, this subsequently shifted to higher but wider altitudes. This finding agrees with studies that found that marmots have widened their ranges and no longer only live on high frigid, subalpine, and alpine meadows at high altitudes [51], and are often found at lower altitudes, around farmland and vegetable fields.

### 4.2. Major Factors That Affect Plague Spatiotemporal Distributions

There was always a strong connection between plague variation and the difference in multifactorial synergy between variables. Climate factors, especially precipitation, are critical for spatial variation. Plague risk increased significantly when PR was between 411.54mm and 493.64mm (Figure 3B). During this study, the cycle changes of plague were in line with precipitation periodical oscillations (10 to 15 years) in the TP over the past several decades [8,52]. In addition, the spatial variation of the plague was consistent with the distribution of precipitation in each stage. For example, regions in Lhasa and the surrounding areas appeared to have more high-risk zones than Qinghai since S2. This was because there was more precipitation and rainy days in Tibet between 1980 and 2013 than in Qinghai [52]. Furthermore, NDVI was also a direct indicator of the diffusion of plague regions. Over the TP, there were many meadow types between northeast and southwest connecting plague niches, which can be easily influenced by precipitation and further cause wider risk distributions. Consequently, in S2, the extensive plague territories were caused by increased participation of NDVI. In fact, these fluctuations in plague and precipitation can be interpreted using the trophic cascade hypothesis. That is, precipitation increases food availability through vegetation-mediated effects, which increases rodent populations and activities, which causes an increase in risk zones [53,54]. However, while these were factors, plague distributions relied on combined effects of a complex synergy of factors rather than just two variables (rainfall and NDVI). For example, in S3, PR rose to 0.83 (Figure 3A) and NDVI to 0.52 to become the third most important factor. However, large-area foci of spatial connectivity did not appear as a result of significantly varying contributions between the variables.

Simultaneously, other inconstant factors such as solar radiation, temperature, soil moisture, and PDSI also impacted plague risk, generating different risk zones. The SR variable had a high value of 244.41 for very high-risk areas (>0.7). The risk threshold of SR was more similar in S4 than in S1, both of which were stages in which the plague epidemic increased. The peak value T for risk shifted rightward along the axes since S1, exhibiting increasing risks with higher temperatures in the subsequent four stages. Indeed, warmer temperatures impact plague through both flea and bacteria (*Y. pestis*) survival [55]; higher temperatures advance the date of returning vegetation following winter, leading to the earlier revival of hosts [56,57]. Recent studies show that as temperatures increase, hosts adapted to cooler climates may suffer an increased risk of infectious disease outbreaks [58]. Furthermore, high irradiance values result in surface drying, combined with the finding that summer soil drying was exacerbated by earlier spring greening [59], which may increase migration for local food shortage, leading to more contact with infectious rodents. Moreover, marmots can suffer declines in body condition because of food decline, tending to exhibit easier defenses by infectious fleas [60]. SM and PDSI were equally variables related to water; thus, they were explained through the influencing mechanism the same as precipitation.

### 4.3. Human Disturbance for Plague Distributions

The reduction of plague because of increased human disturbance can be explained by two factors: first, the 1990s saw serious advancements in the prevention and control of plague, enabling comprehensive monitoring and control of plague among animals, which played an active role in weakening plague intensity (Figure S2) [61]. Second, human disturbance destroys suitable habitats for hosts, resulting in lower abundance thresholds of host populations, thereby decreasing local plague transmission [62]. Unfortunately, other sites with modified habitats (e.g., anthropogenically restored ecological and agricultural sites) may later mediate reservoir abundance and cause changes in rodent and flea community composition, increasing plague risk [35,63]. Additionally, rodents enter sites where the ecology is more susceptible to plague [64]. This explains why plague increased in S5 in our results [65]. Therefore, environmental change should be stressed, and active surveillance programs should be coordinated to prevent the re-emergence of plague [66].

This study had several limitations. There may exist biases in our data as epidemic data were more often collected from general surveillance after human plague was identified. Some sites with adapted environments for plague may thus have not been explored; consequently, our samples may fail to represent all the possible environmental conditions, resulting in a risk underestimation. Additionally, the modelling results were geographical distributions that conformed to the constraint of all the environmental factors based on the epidemic data. However, the barrier restrictions, dispersal disequilibrium, or negative interactions were not considered [67,68], which could result in overestimation of risk [69]. Moreover, we used plague data from the two main hotspots, Qinghai Province and the Tibet Autonomous Region; however, there are risk areas in other provinces, such as the Xinjiang Autonomous Region, which were not considered or modeled (Table 2, Figure 1).

However, these limitations do not invalidate our results and we suggest that our results still demonstrate alterations of the risks and relative influences, which is essential for revealing the long-term effects of climate variation on plague. In the future, projections of plague risk variation under future climate change scenarios are necessary. Homoplastically, when considering human disturbance, it would be invaluable if deeper research could be devoted to understanding the long-term effects on plague risks of eco-relevant exploitation and conservation.

## 5. Conclusions

The study demonstrated differences in the ranges of plague for the five different stages over the TP from 1954–2016. For all stages, the modeled distributions were in agreement with the historical data. The modifications of plague risk regions revealed potential synergistic effects between plague and ecological variability. Specifically, we found that the degree of difference in primary controlling factor compositions changed the spatial pattern of the plague. Generally, climate change-induced wetting generates more favorable sites for plague and may further influence niche differentiation of the Himalayan marmot plague. However, despite the strong connection between climate and plague, we detected significant improvements in human plague dynamics. Our results had advantages on the quantitative description of plague dynamics characterization and generalization of occurrence regularity.

## Figures and Tables

**Table 1:** Environmental variables used in Maxent models for Himalaya plague in the TP.

Data Type	Variables	Biological Relevance	Abbreviation	Units
Topography	DEM	Habitats of hosts: Number of marmot holes is largest at an altitude between 3200–3500 m [33];	E	m
	Distance to river	Field investigation: Almost all marmot holes are around one river;	D	km
	Gravity	Effect in astronomy: Geomagnetism may affect the plague cycle [34];	G	mGal
Vegetation	NDVI	NDVI → Population density: Higher density is often linked to higher prevalence [35];	NDVI	—
Soil	Geochemical landscape	Evolution of *Y. pestis*: Geochemical evolution and biological evolution are a kind of conjugation process→ Persistence of plague [36,37];	GL	—
	Soil type		ST	—
	pH		pH	−log (H+)
	Soil moisture	Vegetation → Population density, migration→ Increased risks [35];	SM	mm
Climate	PDSI	Aridity is significantly associated with ecological risk factors for relapsing plague [36], and drought can control the synchrony of plague outbreaks [36];	PDSI	—
	Precipitation	Phenology [38] → Vegetation → Population density, migration → Increased risks [35];	PR	mm
	Solar Radiation	Governing the surface temperature and hydrologic cycle [39] → Vegetation → Increased risks;	SR	W/m^2^
	Temperature	*Yersinia pestis*: survives for a long time under low temperature conditions [40];Fleas: survival and development → plague persistence [4] Hosts: a prolonged active season [41].	T	°C

**Table 2:** Predicted results at different times over TP.

Phases	The Average Test/Training AUC	Threshold	Average Risk	Areas of Prediction (Thousand km^2^)	Areas of Published Data (Thousand km^2^)
S1	0.93/0.95	0.169	0.041	301.9	99.79
S2	0.90/0.95	0.319	0.055	319.6	—
S3	0.94/0.96	0.218	0.033	247.7	408.38
S4	0.92/0.95	0.259	0.045	303.2	634.49
S5	0.93/0.96	0.180	0.032	263.7	687.04

**Table 3:** Changes of risk areas at different levels of human disturbance.

Change in Human Footprints from 1993 to 2009	Mean Risk in S2	Mean Risk in S3	Mean Risk in S5	Areas with Risk > 0.5 in S2(%)	Areas with Risk > 0.5 in S3(%)	Areas with Risk > 0.5 in S5(%)
−19–−0.01	0.157	0.072	0.068	8.706	1.997	1.198
0	0.094	0.058	0.055	4.999	1.68	1.641
0–2	0.091	0.063	0.052	3.778	1.866	1.588
2–5	0.114	0.060	0.053	6.602	2.117	2.268
5–10	0.107	0.065	0.062	5.310	2.438	3.369
10–20	0.149	0.082	0.077	7.5	2.5	4.167

Average plague risk at different intensity of the human activities.

## Data Availability

Not applicable.

## Supplementary Materials

The following supporting information can be downloaded at: https://www.mdpi.com/article/10.3390/biology11020304/s1, Figure S1: Correlation of environmental variables and occurrence in the five time; Figure S2: Example of risk time series obtained from Maxent results and changes of human footprint; Figure S3: Response curves of soil types and geochemical landscape; Table S1: Environmental variables list; Table S2: The classification of geochemical landscape.

## References

[1] Jones B.A., Grace D., Kock R., Alonso S., Rushton J., Said M.Y., McKeever D., Mutua F., Young J., McDermott J. (2013). Zoonosis emergence linked to agricultural intensificationand environmental change. Proc. Natl. Acad. Sci. USA.

[2] Rasmussen S., Allentoft M., Nielsen K., Orlando L., Sikora M., Sjögren K.-G., Pedersen A.G., Schubert M., Van Dam A., Kapel C. (2015). Early divergent strains of *Yersinia pestis* in Eurasia 5,000 years ago. Cell.

[3] Reijniers J., Davis S., Begon M., Heesterbeek J.A., Ageyev V.S., Leirs H. (2012). A curve of thresholds governs plague epizootics in Central Asia. Ecol. Lett..

[4] Wimsatt J., Biggins D.E. (2009). A review of plague persistence with special emphasis on fleas. J. Vector. Borne. Dis..

[5] Weinhold B. (2000). Plague linked to precipitation. Environ. Health Perspect..

[6] Liu X.D., Chen B.D. (2000). Climatic warming in the Tibetan Plateau during recent decades. Int. J. Climatol..

[7] Wang X., Wei X., Song Z., Wang M., Xi J., Liang J., Liang Y., Duan R., Tian K., Zhao Y. (2017). Mechanism study on a plague outbreak driven by the construction of a large reservoir in southwest china (surveillance from 2000–2015). PLoS Negl. Trop. Dis..

[8] Xu X., Cui Y., Xin Y., Yang X., Zhang Q., Jin Y., Zhao H., He J., Jin X., Li C. (2018). Genetic diversity and spatial-temporal distribution of *Yersinia pestis* in Qinghai Plateau, China. PLoS Negl. Trop. Dis..

[9] Dai R., Qi M., Xiong H., Yang X., He J., Zhang Z., Yang H., Jin J., Li X., Xin Y. (2019). Serological Epidemiological Investigation of Tibetan Sheep (Ovis aries) Plague in Qinghai, China. Vector. Borne. Zoonotic. Dis..

[10] Walsh M., Haseeb M.A. (2015). Modeling the ecologic niche of plague in sylvan and domestic animal hosts to delineate sources of human exposure in the western United States. PeerJ.

[11] Aguilar M., Lado C. (2012). Ecological niche models reveal the importance of climate variability for the biogeography of protosteloid amoebae. ISME J..

[12] Qian Q., Zhao J., Fang L., Zhou H., Zhang W., Wei L., Yang H., Yin W., Cao W., Li Q. (2014). Mapping risk of plague in Qinghai-Tibetan Plateau, China. BMC Infect. Dis..

[13] Neerinckx S., Peterson A.T., Gulinck H., Deckers J., Kimaro D., Leirs H. (2010). Predicting Potential Risk Areas of Human Plague for the Western Usambara Mountains, Lushoto District, Tanzania. Am. J. Trop. Med. Hyg..

[14] Giles J., Peterson A.T., Almeida A. (2011). Ecology and Geography of Plague Transmission Areas in Northeastern Brazil. PLoS Negl. Trop. Dis..

[15] Tian L. (2018). Relationship between environmental factors and the spatial distribution of Spermophilus dauricus during 2000–2015 in China. Int. J. Biometeorol..

[16] Lu L., Ren Z., Yue Y., Yu X., Lu S., Li G., Li H., Wei J., Liu J., Mu Y. (2016). Niche modeling predictions of the potential distribution of Marmota himalayana, the host animal of plague in Yushu County of Qinghai. BMC Public Health.

[17] Stenseth N.C., Samia N.I., Viljugrein H., Kausrud K.L., Begon M., Davis S., Leirs H., Dubyanskiy V.M., Esper J., Ageyev V.S. (2006). Plague dynamics are driven by climate variation. Proc. Natl. Acad. Sci. USA.

[18] Xu L., Liu Q., Stige L.C., Ben Ari T., Fang X., Chan K.-S., Wang S., Stenseth N.C., Zhang Z. (2011). Nonlinear effect of climate on plague during the third pandemic in China. Proc. Natl. Acad. Sci. USA.

[19] Redding D.W., Atkinson P.M., Cunningham A.A., Lo Iacono G., Moses L.M., Wood J.L., Jones K.E. (2019). Impacts of environmental and socio-economic factors on emergence and epidemic potential of Ebola in Africa. Nat. Commun..

[20] Ben Ari T., Neerinckx S., Gage K.L., Kreppel K., Laudisoit A., Leirs H., Stenseth N.C. (2011). Plague and climate: Scales matter. PLoS Pathog..

[21] Seaborn T., Goldberg C.S., Crespi E.J. (2021). Drivers of distributions and niches of North American cold-adapted amphibians: Evaluating both climate and land use. Ecol. Appl..

[22] Holt A.C., Salkeld D.J., Fritz C.L., Tucker J.R., Gong P. (2009). Spatial analysis of plague in California: Niche modeling predictions of the current distribution and potential response to climate change. Int. J. Health Geogr..

[23] Ben-Ari T., Neerinckx S., Agier L., Cazelles B., Xu L., Zhang Z., Fang X., Wang S., Liu Q., Stenseth N.C. (2012). Identification of Chinese plague foci from long-term epidemiological data. Proc. Natl. Acad. Sci. USA.

[24] Global Multi-resolution Terrain Elevation Data 2010 courtesy of the U.S. Geological Survey. https://topotools.cr.usgs.gov/gmted_viewer/viewer.htm.

[25] Xu x. Dataset of China River Basin and River Network Based on DEM Extraction. https://www.resdc.cn/data.aspx?DATAID=226.

[26] Global Gravity Field Model. https://www.space.dtu.dk/english/Research/Scientific_data_and_models/Global_Marine_Gravity_Field.

[27] Landsat Collection Courtesy of the, U.S. Geological Survey. https://earthexplorer.usgs.gov.

[28] Map of Chemico-Geographic Landscape of China (2000). The Atlas of Plague and Its Environment in the People’s Republic of China.

[29] Soil pH in H2O at 6 Standard Depths (0, 10, 30, 60, 100 and 200 cm) at 250 m Resolution. https://zenodo.org/record/2525664#.YgX_zN9BySk.

[30] FAO/IIASA/ISRIC/ISSCAS/JRC (2012). Harmonized World Soil Database (version 1.2). FAO, Rome, Italy and IIASA, Laxenburg, Austria. https://previous.iiasa.ac.at/web/home/research/researchPrograms/water/HWSD.html.

[31] Abatzoglou J.T., Dobrowski S.Z., Parks S.A., Hegewisch K.C. (2018). TerraClimate, a high-resolution global dataset of monthly climate and climatic water balance from 1958–2015. Sci. Data..

[32] Venter O., Sanderson E.W., Magrach A., Allan J.R., Beher J., Jones K.R., Possingham H.P., Laurance W.F., Wood P., Fekete B.M. (2016). Global terrestrial Human Footprint maps for 1993 and 2009. Sci. Data..

[33] Gao M., Li X., Cao C., Zhang H., Li Q., Zhou H., He Q., Xu M., Zhao J., Zheng S. (2010). Spatial prediction and analysis of Himalayan marmot plague natural epidemic foci in China based on HJ-1 satellite data. Sci. China-Earth Sci..

[34] Zaporozhan V., Ponomarenko A. (2010). Mechanisms of Geomagnetic Field Influence on Gene Expression Using Influenza as a Model System: Basics of Physical Epidemiology. Int. J. Env. Res. Public Health.

[35] Mendoza H., Rubio A.V., Garcia-Pena G.E., Suzan G., Simonetti J.A. (2020). Does land-use change increase the abundance of zoonotic reservoirs? Rodents say yes. Eur. J. Wildl. Res..

[36] Barbieri R., Texier G., Keller C., Drancourt M. (2020). Soil salinity and aridity specify plague foci in the United States of America. Sci. Rep..

[37] Malek M.A., Bitam I., Levasseur A., Terras J., Gaudart J., Azza S., Flaudrops C., Robert C., Raoult D., Drancourt M. (2017). *Yersinia pestis* halotolerance illuminates plague reservoirs. Sci. Rep..

[38] Shen M., Piao S., Cong N., Zhang G., Jassens I.A. (2015). Precipitation impacts on vegetation spring phenology on the Tibetan Plateau. Glob. Chang. Biol..

[39] Naud C.M., Rangwala I., Xu M., Miller J.R. (2015). A Satellite View of the Radiative Impact of Clouds on Surface Downward Fluxes in the Tibetan Plateau. J. Appl. Meteorol. Clim..

[40] Munyenyiwa A., Zimba M., Nhiwatiwa T., Barson M. (2019). Plague in Zimbabwe from 1974 to 2018: A review article. PLoS Negl. Trop. Dis..

[41] Tanser F.C., Sharp B., Le Sueur D. (2003). Potential effect of climate change on malaria transmission in Africa. Lancet.

[42] Liu Y., Huang P., Lin F., Yang W., Gaisberger H., Christopher K., Zheng Y. (2019). MaxEnt modelling for predicting the potential distribution of a near threatened rosewood species (Dalbergia cultrata Graham ex Benth). Ecol. Eng..

[43] Wan J., Qi G.J., Ma J., Ren Y., Wang R., McKirdy S. (2020). Predicting the potential geographic distribution of Bactrocera bryoniae and Bactrocera neohumeralis (Diptera: Tephritidae) in China using MaxEnt ecological niche modeling. J. Integr. Agric..

[44] Potter K.A., Arthur Woods H., Pincebourde S. (2013). Microclimatic challenges in global change biology. Glob. Chang. Biol..

[45] Phillips S.J., Anderson R.P., Schapire R.E. (2006). Maximum entropy modeling of species geographic distributions. Ecol. Model..

[46] Phillips S.J., Dudik M. (2008). Modeling of species distributions with Maxent: New extensions and a comprehensive evaluation. Ecography.

[47] Phillips S.J., Anderson R.P., Dudik M., Schapire R.E., Blair M.E. (2017). Opening the black box: An open-source release of Maxent. Ecography.

[48] Wang G., Wang C., Guo Z., Dai L., Wu Y., Liu H., Li Y., Chen H., Zhang Y., Zhao Y. (2020). Integrating Maxent model and landscape ecology theory for studying spatiotemporal dynamics of habitat: Suggestions for conservation of endangered Red-crowned crane. Ecol. Indic..

[49] Xu N., Meng F., Zhou G., Li Y., Wang B., Lu H. (2020). Assessing the suitable cultivation areas for Scutellaria baicalensis in China using the Maxent model and multiple linear regression. Biochem. Syst. Ecol..

[50] Nikol–skii A.A., Ulak A. (2006). Key factors determining the ecological niche of the Himalayan marmot, Marmota himalayana Hodgson (1841). Russ. J. Ecol..

[51] Li D. (1995). Preliminary Observation of Marmot Habitat Movement. J. Med. Pest. Control..

[52] Han Y., Ma W., Wang B., Ma Y., Tian R. (2017). Climatic characteristics of rainfall change over the Qinghai- Tibetan Plateau from 1980 to 2013. Plateau. Meteor..

[53] Ben Ari T., Gershunov A., Gage K.L., Snäll T., Ettestad P., Kausrud K.L., Stenseth N.C. (2008). Human plague in the USA: The importance of regional and local climate. Biol. Lett..

[54] Davis S., Begon M., De Bruyn L., Ageyev V.S., Klassovskiy N.L., Pole S.B., Viljugrein H., Stenseth N.C., Leirs H. (2004). Predictive thresholds for plague in Kazakhstan. Science.

[55] Russell R.E., Abbott R.C., Tripp D.W., Rocke T.E. (2018). Local factors associated with on-host flea distributions on prairie dog colonies. Ecol. Evol..

[56] Eisen R.J., Gage K.L. (2009). Adaptive strategies of *Yersinia pestis* to persist during inter-epizootic and epizootic periods. Vet. Res..

[57] Eads D.A., Abbott R.C., Biggins D.E., Rocke T.E. (2020). Flea Parasitism and Host Survival in a Plague-Relevant System: Theoretical and Conservation Implications. J. Wildl. Dis..

[58] Cohen J.M., Sauer E.L., Santiago O., Spencer S., Rohr J.R. (2020). Divergent impacts of warming weather on wildlife disease risk across climates. Science.

[59] Lian X., Piao S., Li L.Z.X., Li Y., Huntingford C., Ciais P., Cescatti A., Janssens I.A., Peñuelas J., Buermann W. (2020). Summer soil drying exacerbated by earlier spring greening of northern vegetation. Sci. Adv..

[60] Eads D.A., Biggins D.E., Xu L., Liu Q. (2016). Plague cycles in two rodent species from China: Dry years might provide context for epizootics in wet years. Ecosphere.

[61] Wang H., Wang G., Wang Z., Li C., Li M. (2004). Retrospection and present state of plague prevention and control in Qinghai province about 50 years. Zhonghua Difangbingxue Zazhi.

[62] Wilschut L.I., Addink E.A., Heesterbeek H., Heier L., Laudisoit A., Begon M., de Jong S.M. (2013). Potential corridors and barriers for plague spread in central Asia. Int. J. Health. Geogr..

[63] McCauley D.J., Dirzo R., Young H.S., Salkeld D.J., Gaffikin L., Barry M., Lambin E.F., Helgen K.M., Eckerlin R.P., Makundi R. (2015). Effects of Land Use on Plague (*Yersinia pestis*) Activity in Rodents in Tanzania. Am. J. Trop. Med. Hyg..

[64] Russell R.E., Tripp D.W., Rocke T.E. (2019). Differential plague susceptibility in species and populations of prairie dogs. Ecol. Evol..

[65] Cabanel N., Leclercq A., Chenal-Francisque V., Annajar B., Rajerison M., Bekkhoucha S., Bertherat E., Carniel E. (2013). Plague Outbreak in Libya, 2009, Unrelated to Plague in Algeria. Emerg. Infect. Dis..

[66] Duplantier J.M., Duchemin J.B., Chanteau S., Carniel E. (2005). From the recent lessons of the Malagasy foci towards a global understanding of the factors involved in plague reemergence. Vet. Res..

[67] Tang C.Q., Matsui T., Ohashi H., Dong Y.F., Momohara A., Herrando-Moraira S., Qian S., Yang Y., Ohsawa M., Luu H.T. (2018). Identifying long-term stable refugia for relict plant species in East Asia. Nat. Commun..

[68] Soberon J., Peterson A.T. (2005). Interpretation of Models of Fundamental Ecological Niches and Species’ Distributional Areas. Biodiv. Inf..

[69] Collinge S.K., Johnson W.C., Ray C., Matchett R., Grensten J., Cully J.F., Martin A.P. (2005). Landscape structure and plague occurrence in black-tailed prairie dogs on grasslands of the western USA. Landsc. Ecol..

