# Supplementary material for: Spatiotemporal Variations of Plague Risk in the Tibetan Plateau from 1954–2016
Source: Biology (Basel). 2022 Feb 13;11(2):304. doi: 10.3390/biology11020304 (PMC8869688; doi:10.3390/biology11020304)
Supplement: Supplementary file 1 [file biology-11-00304-s001.zip › biology-1544821-supplementary.pdf]

Supplementary Materials

Spatiotemporal Variations of Plague Risk in the Tibetan Plateau from 1954–2016

Xing Yuan [1,2], Linsheng Yang [1,2], Hairong Li [1,2,*] and Li Wang [1]

1 Key Laboratory of Land Surface Pattern and Simulation, Institute of Geographic Sciences and Natural Resources Research, Chinese Academy of Sciences, Beijing 100101, China; yuanx.19b@igsnrr.ac.cn (X.Y.); yangls@igsnrr.ac.cn (L.Y.); wangli@igsnrr.ac.cn (L.W.)

2 College of Resources and Environment, University of Chinese Academy of Sciences, Beijing 100049, China

* Correspondence: **lihr@igsnrr.ac.cn**

Table of Contents

Table S1. Environmental variables list.
Figure S1. Correlation of environmental variables and occurrence in the five time.
Figure S2. Example of risk time series obtained from Maxent results and changes of human footprint.
Figure S3. Response curves of soil types and geochemical landscape.
Table S2. The classification of geochemical landscape.

Table S1. Environmental Variables. The variables in bold were ultimately selected (12/22).

**Dataset**	**Description**	**Unit**
Topography		
**Dem**	**Elevation**	**m**
RDLS	Topographic relief	-
**GRAVITY**	**Global Gravity Field**	**mGal**
Vegetation		
**NDVI_YEARLY_MEDIAN**	**Calculated using GLS1975 and Landsat5\7\8**	-
Climate		
**PDSI_YEARLY_MEAN**	**Palmer Drought Severity Index, capture the basic effect of global warming on drought through changes in potential evapotranspiration**	**-**
**PR_YEARLY_MEAN**	**Precipitation accumulation yearly**	**mm**
**SM_YEARLY_MEAN**	**Soil moisture, derived using a one-dimensional soil water balance model**	**mm**
PET_GROWING_MEAN	Reference evapotranspiration (ASCE Penman-Montieth)	mm
WS_GROWING_MEAN	Wind-speed at 10m	m/s
PR_GROWING_MEAN	Precipitation accumulation between monthly 4–10	mm
**SR_GROWING_MEAN**	**Downward Solar Radiation Flux at the Surface during growing season**	W/m^2
T4-**T10**_MONTHLY_**MEAN**	**Min temperature**	**°C**
Soil		
**Soil type**	**FAO90 classification**	-
**Geochemical landscape**	**Geochemical characteristics of natural landscape**	-
**pH**	**Soil pH in H_2O at 200cm depth**	**-log (H^+)**
Others		
**Distance to River**	**Euclidean distances**	**km**

The data sources can be found in the articles.

Figure S1. Correlation of environmental variables and occurrence in the five time.

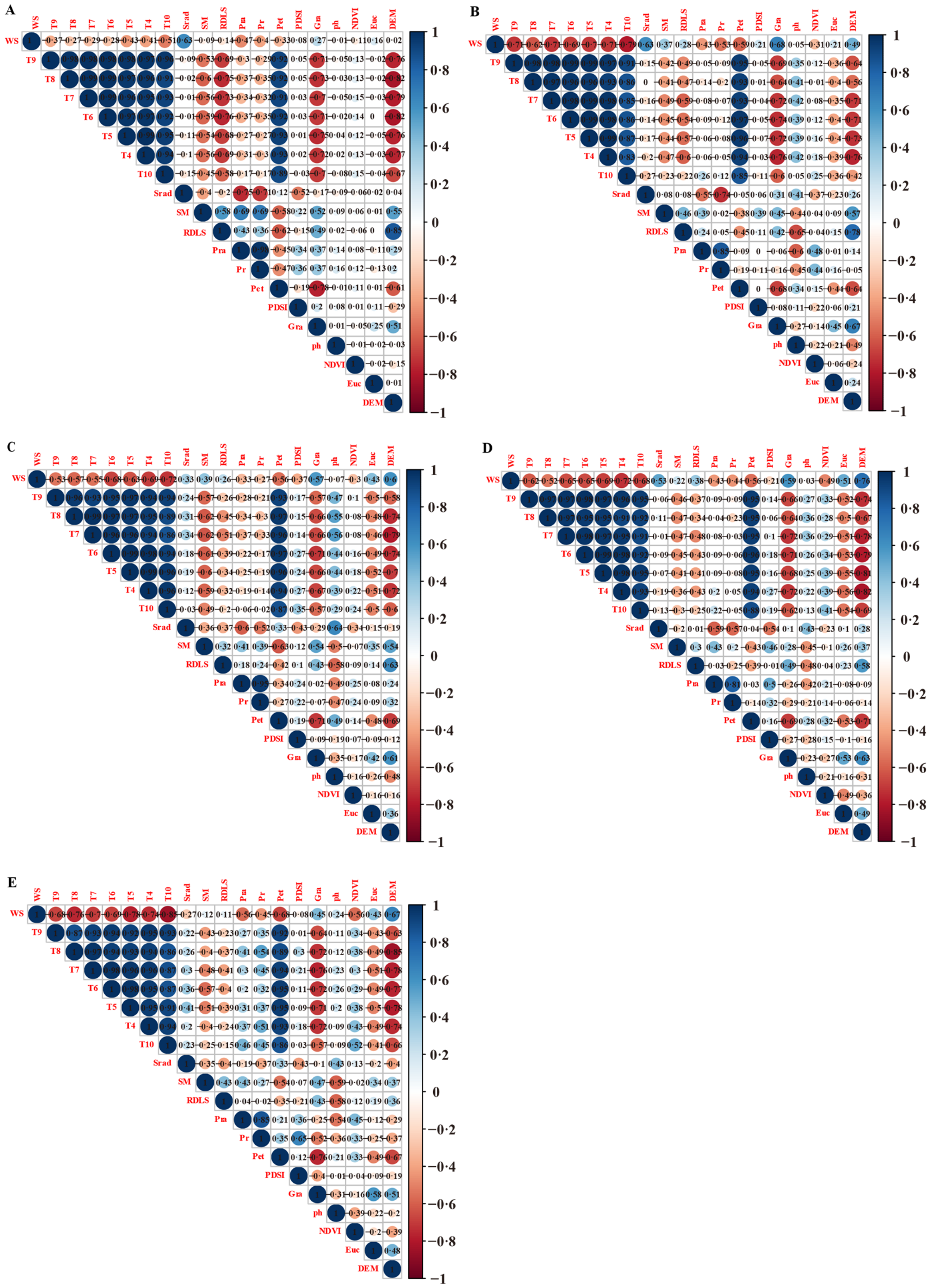

Figure S1**A–E** were from Pearson's correlation analysis of S1, S2, S3, S4, and S5 respectively. Blue represents positive correlation, and red represents negative correlation. The larger the circle, the stronger the correlation.

Figure S2. Example of risk time series obtained from Maxent results and changes of human footprint.

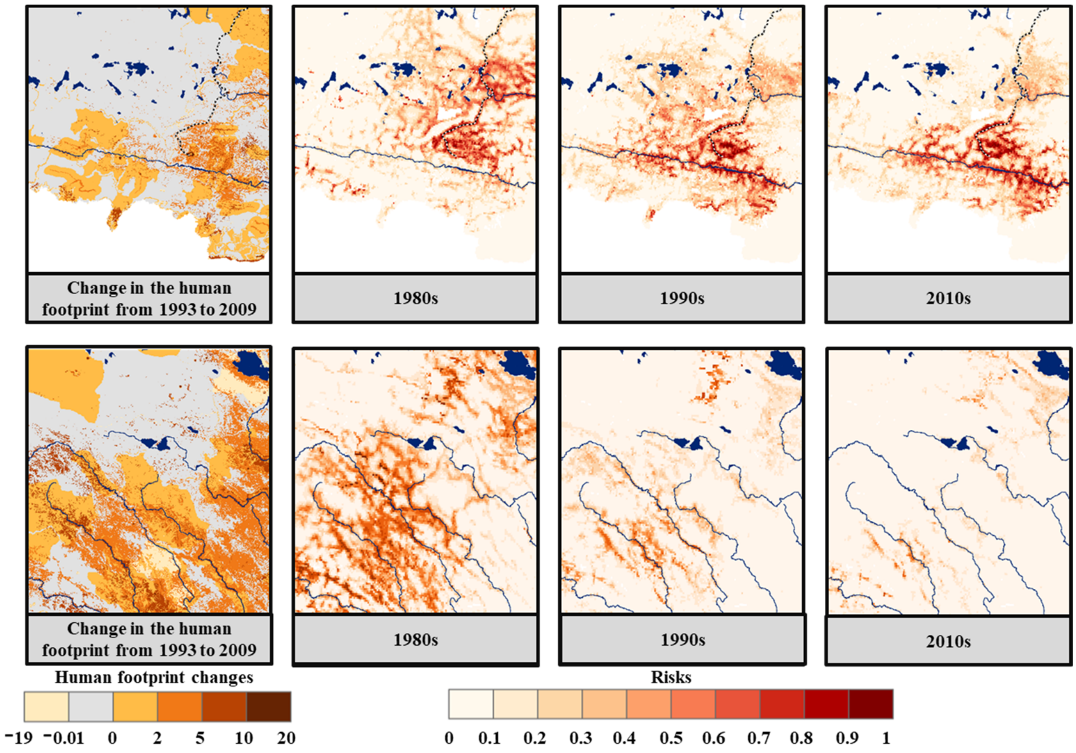

Two distinct typical areas were chosen to display how human activity may change plague risks. Plots on the left show the change in the human footprint from 1993 to 2009. The three on the right are changes in plague risk of S2, S3 and S5 within a subset of TP. Plots in the first line locate in the YNL region (Yarlung Zangbo River, the Nyangqu and the Lhasa River Region) in Tibet, with ecological protection. The second line locate around southwestern Qinghai Lake and Yushu Tibetan Autonomous Prefecture with less ecological protection.

Figure S3. Response curves of soil types and geochemical landscape.

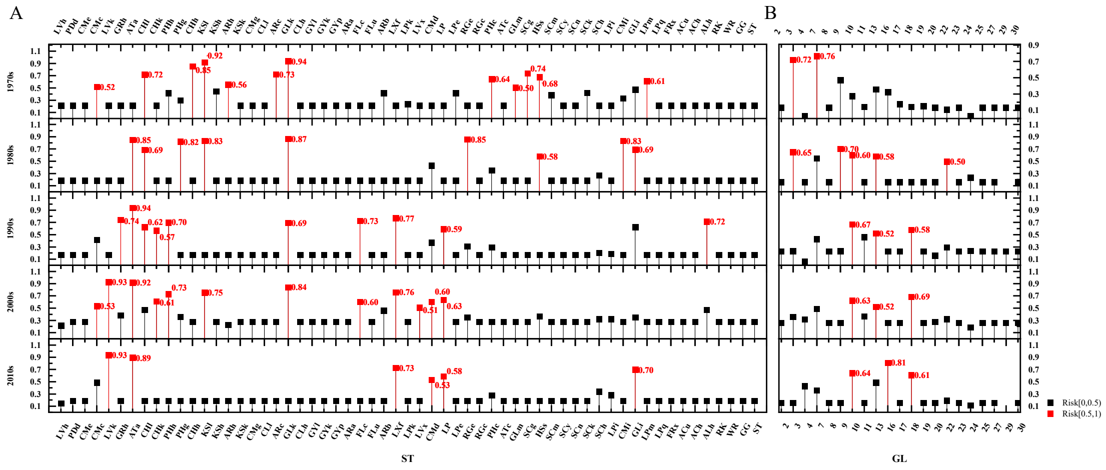

Figure 3**A** shows the soil type in Himalayan marmot foci, the classification is based on FAO 90. The detailed list of FAO90 soil types can be found on pages 24 and 25 of the pdf (https://www.fao.org/3/aq361e/aq361e.pdf). Figure 3**B** shows the geochemical landscape in Himalayan marmot foci and their types are listed in the following table 2.

Table S2. The classification of geochemical landscape.

Code	Type
2	Temperate forest leaching landscape
3	Temperate humid steppe shallow leaching accumulation landscape
4	High-frigid desert surface-accumulation landscape
7	Temperate dry steppe shallow leaching-accumulation landscape
8	Sandy land and sandy desert landscape
9	Temperate desert steppe shallow leaching-accumulation landscape
10	Subalpine meadow weak leaching-accumulation landscape
11	Temperate desert surface accumulation landscape
13	Alpine meadow weak leaching-accumulation landscape
16	Temperate desert steppe shallow leaching-accumulation landscape pile
17	Warm temperate desert surface accumulation landscape
18	Subalphine steppe weak leaching-accumulation landscape
19	Salt accumulation landscape
20	Warm temperate forest leaching landscape
22	Warm temperate dry forest leaching landscape
23	Warm temperate forest steppe laeching landscape
24	Alpine steppe weak leaching-accumulation landscape
25	North subtopical forest leaching landscape
27	Central subtropical forest strong leaching-accumulation landscape
29	Central subtropical forest strong leaching-accumulation landscape pile
30	South subtropical forest strong leaching-accumulation landscape
